# Salicylic Acid in Rheumatism

**Published:** 1884-04-20

**Authors:** C. Barlow

**Affiliations:** Eaton, Ill.


### SALICYLIC ACID IN RHEUMATISM.

BY C. BARLOW, M.D., EATON, ILL.

In acute and subacute rheumatism I believe we have no more potent remedy than salicylic acid. My first experience with it led me to doubt its utility. I first administered it in a few cases in which I did not observe any beneficial results. In those cases I only gave five or six grains every three or four hours. I then increased the dose and gave it at shorter intervals, and the result was more satisfactory. About this time the subject came up for discussion before our medical society, and Dr. S. D. Meserve remarked that he considered salicylic acid just as much a specific for acute and subacute rheumatism as quinine is for malaria. He stated that in order to obtain satisfactory results it should be given in sufficient doses, and at short enough intervals to bring the patient thoroughly under its influence. Then prolong the time between doses; but give enough to keep patient well under its influence until recovery is complete. He advised it to be given in combination with acetate of potash, as follows:

℞ Salicylic acid, Acetate of potash. } ........................aa ʒ ij,

Water........................................℥ jss.

M. S. Teaspoonful every two hours.

After a day or two he extended the time to three or four hours. In the above mixture there is ten grains to the dose. He stated that some patients would require fifteen grains every two hours at first. Now, I have adopted this plan of treatment in quite a number of cases and have not been disappointed in a single instance. The last patient I treated for rheumatism was a small boy. All the joints were affected from the hips down; also his wrists. It was a severe case, and was under treatment only about one week. Recovery complete. There was marked improvement in this case in twenty-four hours after the first dose was given. I had an attack of rheumatism, myself, in right knee, last spring, in which I tried the salicylic acid treatment. The inflamed joint was swollen and very painful. Temperature 103°, pulse 100. Took fifteen grains of the acid in solution with as much acetate of potash, every two hours during the first forty-eight hours, in two or three ounces of sweetened water. There was some improvement at the end of twenty-four hours, and very marked relief in forty-eight hours. I then lengthened the time between doses to four hours. Continued to improve, and on the fourth day I visited a patient six miles from home; atmosphere damp, and raining part of the time while on the road. Was taken worse before reaching home, and had to take morphine to relieve pain. Took the medicine two hours apart during the night, and was much better next day, when I took medicine four hours apart. Continued to improve, had a good night, and was much better next day until late in the evening, when I visited another patient, and was caught out in a heavy rain. I got wet and took another relapse that night; but two or three doses of the medicine two hours apart were aufficient to relieve urgent symptoms, and after a day or two more I was able to ride again. I now attended to my practice regularly, but experienced one or two more slight relapses, which were relieved by increasing the medicine as before. This attack lasted about three weeks, and afforded a splendid opportunity to test the efficacy of the treatment employed. Each relapse was promptly checked by increasing the medicine, and I believe that if I had avoided exposure the recovery would have been complete in a week or ten days. The only disagreeable effect I noticed from the medicine was a *tinnitus aurium* very much like that produced by quinine.—*Ther. Gaz.*

